# Supplementary material for: Reconfigurable Terahertz Multi‐Harmonic Dual‐Combs
Source: Nanophotonics. 2026 Sep 2;15(17):e70281. doi: 10.1002/nap2.70281 (PMC13672152; doi:10.1002/nap2.70281)
Supplement: Supplementary file 1 [file NAP2-15-e70281-s001.pdf]

Supporting Information

Reconfigurable Terahertz Multi-Harmonic Dual-Combs

Xuhong Ma*, **, Zhiwei Qin*, Xianglong Bi, Ziping Li, Wenjian Wan, Binbin Liu, Guibin Liu, Yue Pan, Yanming Lu, Hua Li**

Dr. X. Ma, Z. Qin, X. Bi, Dr. Z. Li, Dr. W. Wan, Dr. B. Liu, G. Liu, Y. Pan, Y. Lu, Prof. H. Li
National Key Laboratory of Materials for Integrated Circuits and Key Laboratory of Terahertz Solid State Technology, Shanghai Institute of Microsystem and Information Technology, Chinese Academy of Sciences, 865 Changning Road, Shanghai 200050, China

Z. Qin, X. Bi, Dr. Z. Li, Dr. W. Wan, G. Liu, Y. Pan, Y. Lu, Prof. H. Li
Center of Materials Science and Optoelectronics Engineering, University of Chinese Academy of Sciences, Beijing 100049, China

Dr. X. Ma, Dr. B. Liu
Chongqing Key Laboratory of Precision Optics, Chongqing Institute of East China Normal University, Chongqing 401120, China

*Corresponding authors.
E-mail: maxuhong@mail.sim.ac.cn, hua.li@mail.sim.ac.cn

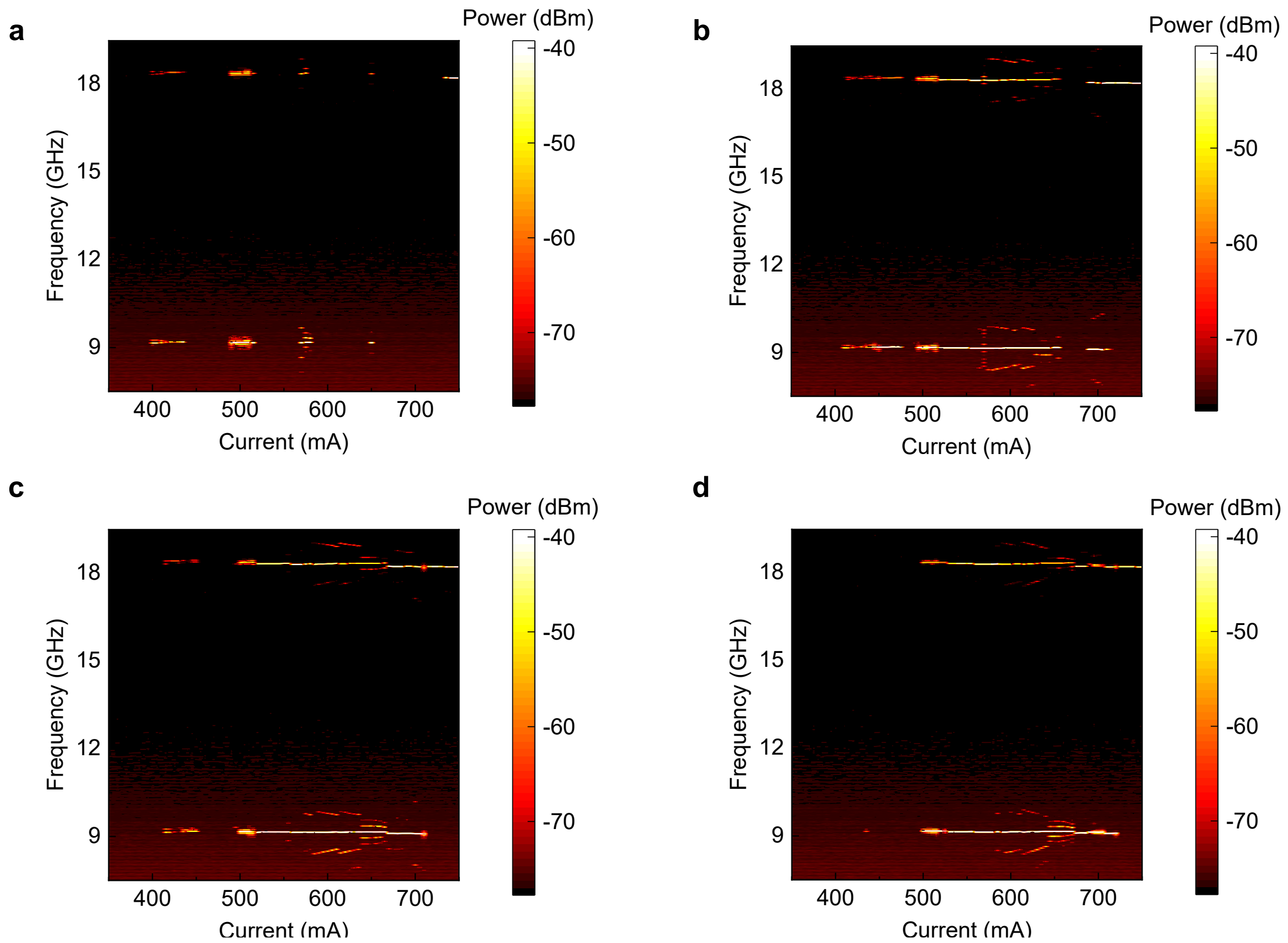

Figure S1: Intermode beatnote maps of QCL1 measured under different operating conditions. Panels **a–d** show the results measured under free-running, 20 K, 25 K, and 30 K conditions, respectively, with a resolution bandwidth (RBW) of 100 kHz and a video bandwidth (VBW) of 10 kHz. Beatnote features are observed around 1 × FSR (~9 GHz) and 2 × FSR (~18 GHz), and their evolution with current and temperature reveals different stable operating regions associated with the fundamental comb and harmonic comb states. These results indicate that the comb-operation window and intermode beatnote stability of QCL1 are strongly influenced by temperature.

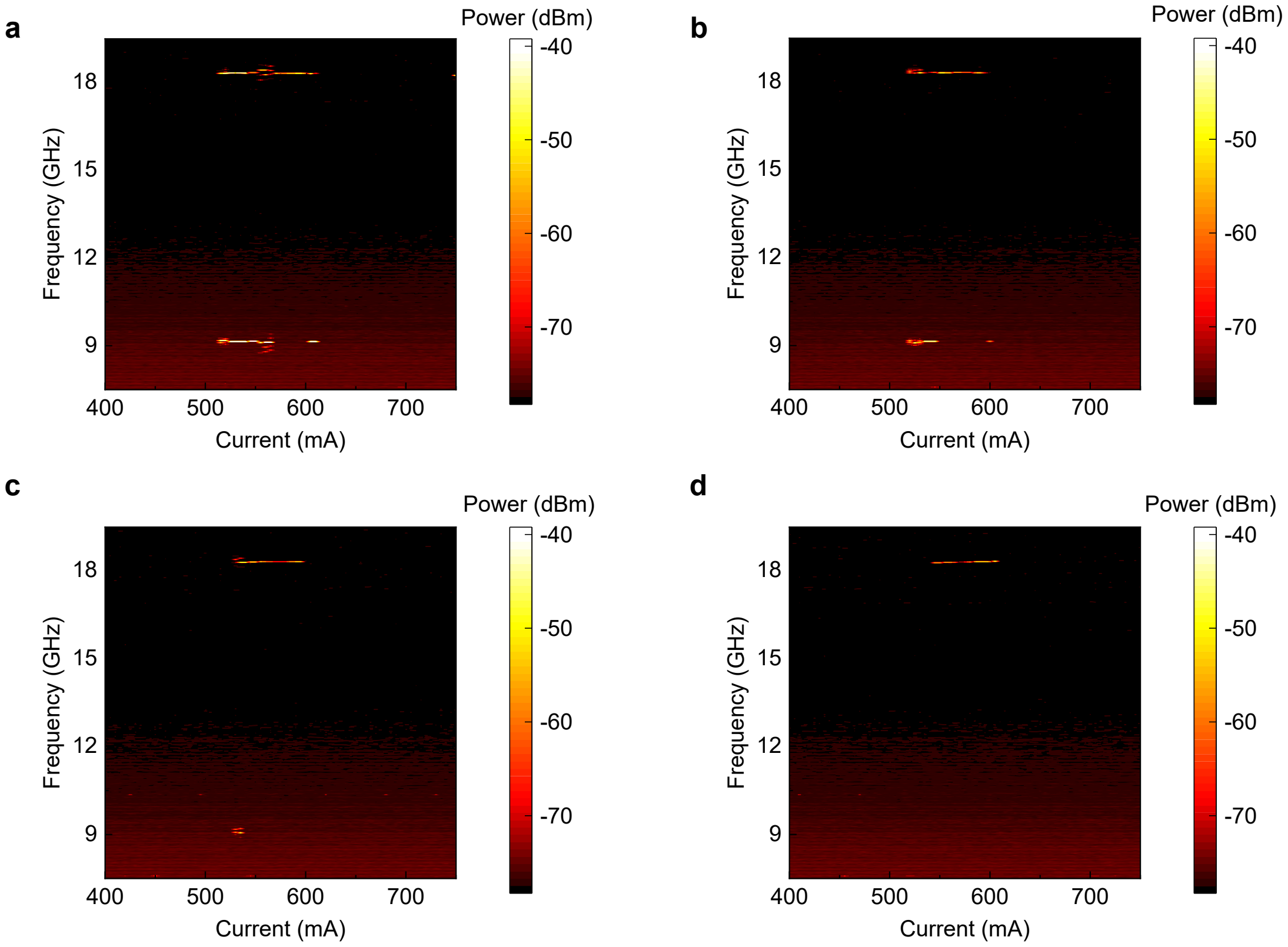

Figure S2: Intermode beatnote maps of QCL2 measured under different conditions. Panels **a–d** show the results obtained under free-running, 20 K, 25 K, and 30 K, respectively, with an RBW of 100 kHz and a VBW of 10 kHz. The narrow beatnote features near 9 GHz (1 × FSR) and 18 GHz (2 × FSR) indicate different comb-operation regimes under different thermal and bias conditions and reveal a pronounced temperature dependence.

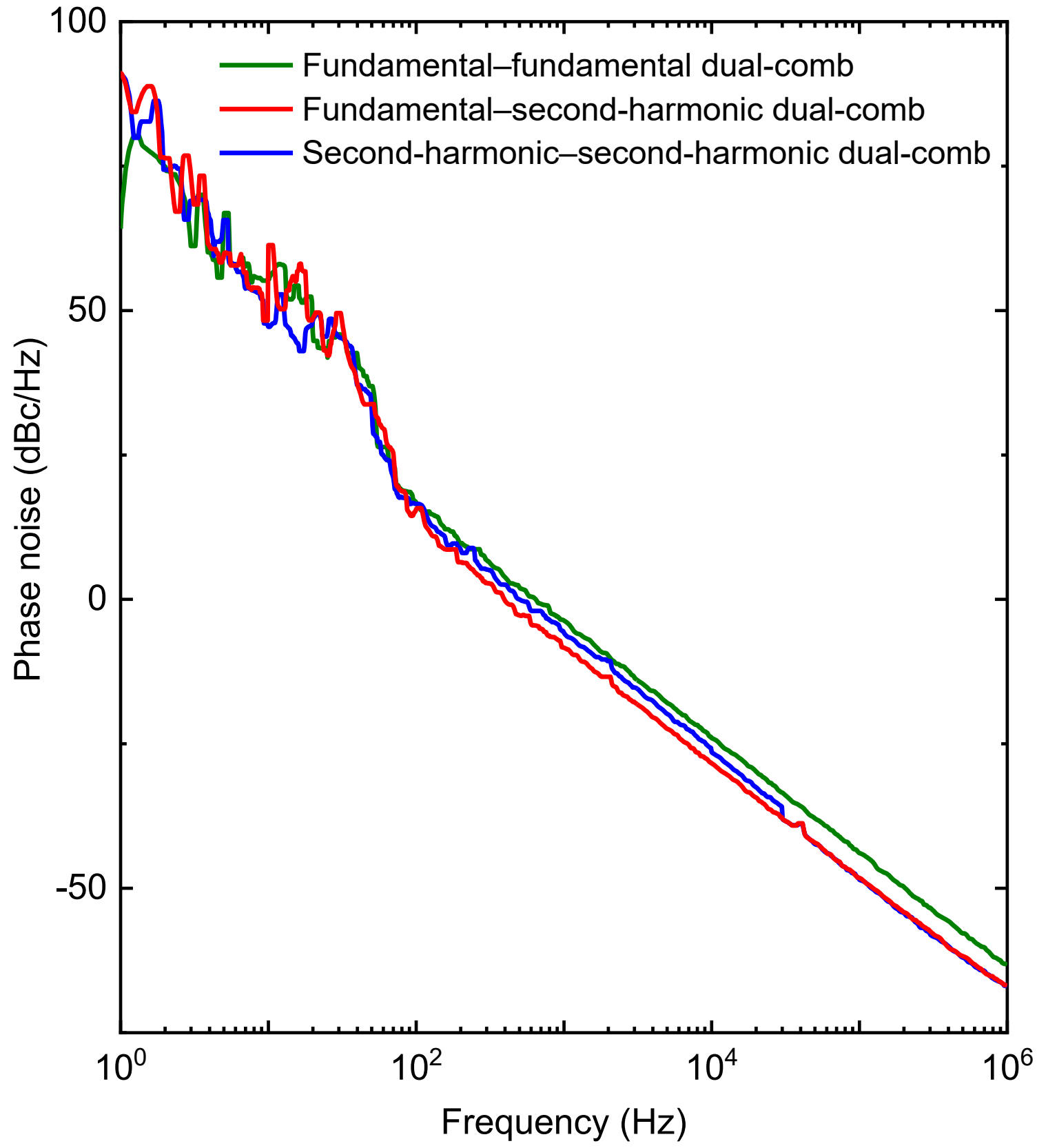

Figure S3: Comparison of the phase noise spectra of a representative multi-heterodyne mode under three dual-comb operating states. The traces correspond to the modes marked by the star symbol in Figures 3–5 of the main text, representing the fundamental–fundamental dual-comb, fundamental–second-harmonic dual-comb, and second-harmonic–second-harmonic dual-comb configurations, respectively. The three spectra exhibit overall similar trends and comparable noise levels over the entire measurement range, indicating that the representative dual-comb modes possess similar frequency stability despite the different harmonic-order combinations and RF frequency-mapping relations involved in the three operating regimes. This observation is consistent with the linewidth analysis of the long-term max-hold spectra presented in the main text, which shows that coherent dual-comb operation with comparable long-term stability is maintained across the different harmonic configurations.

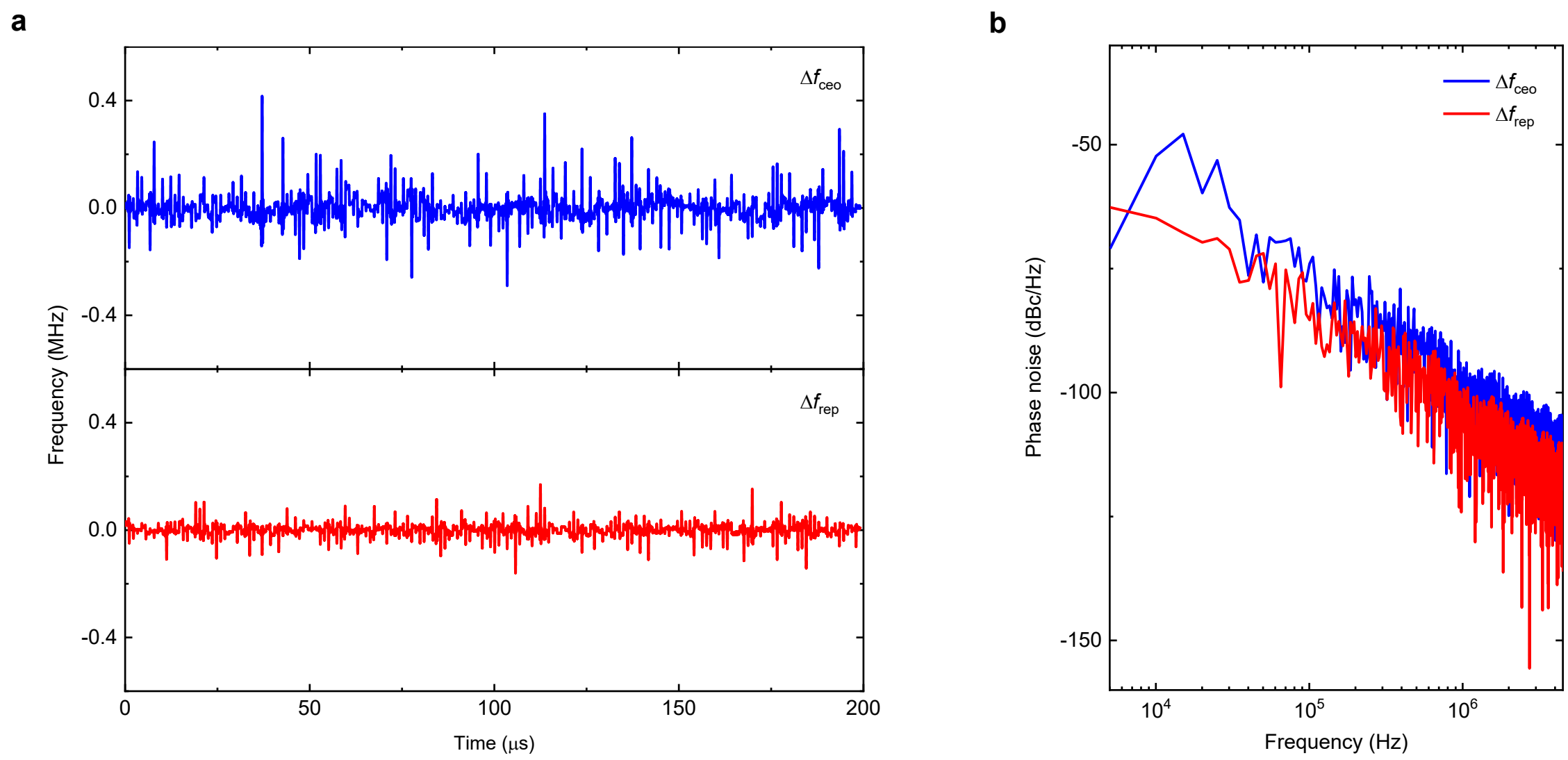

Figure S4: Results obtained from a single-shot acquisition window of 200 μs for the fundamental–fundamental dual-comb state. **a** Instantaneous frequency-fluctuation traces extracted from the raw dual-comb time-domain interferogram, where the upper trace corresponds to the carrier-envelope offset frequency difference Δf_{ceo} and the lower trace corresponds to the repetition frequency difference Δf_{rep}. **b** Corresponding single-sideband phase noise spectra derived from these frequency fluctuations. These results characterize the residual frequency and timing jitter associated with Δf_{ceo} and Δf_{rep} prior to phase correction and provide the basis for the subsequent compensation procedure.

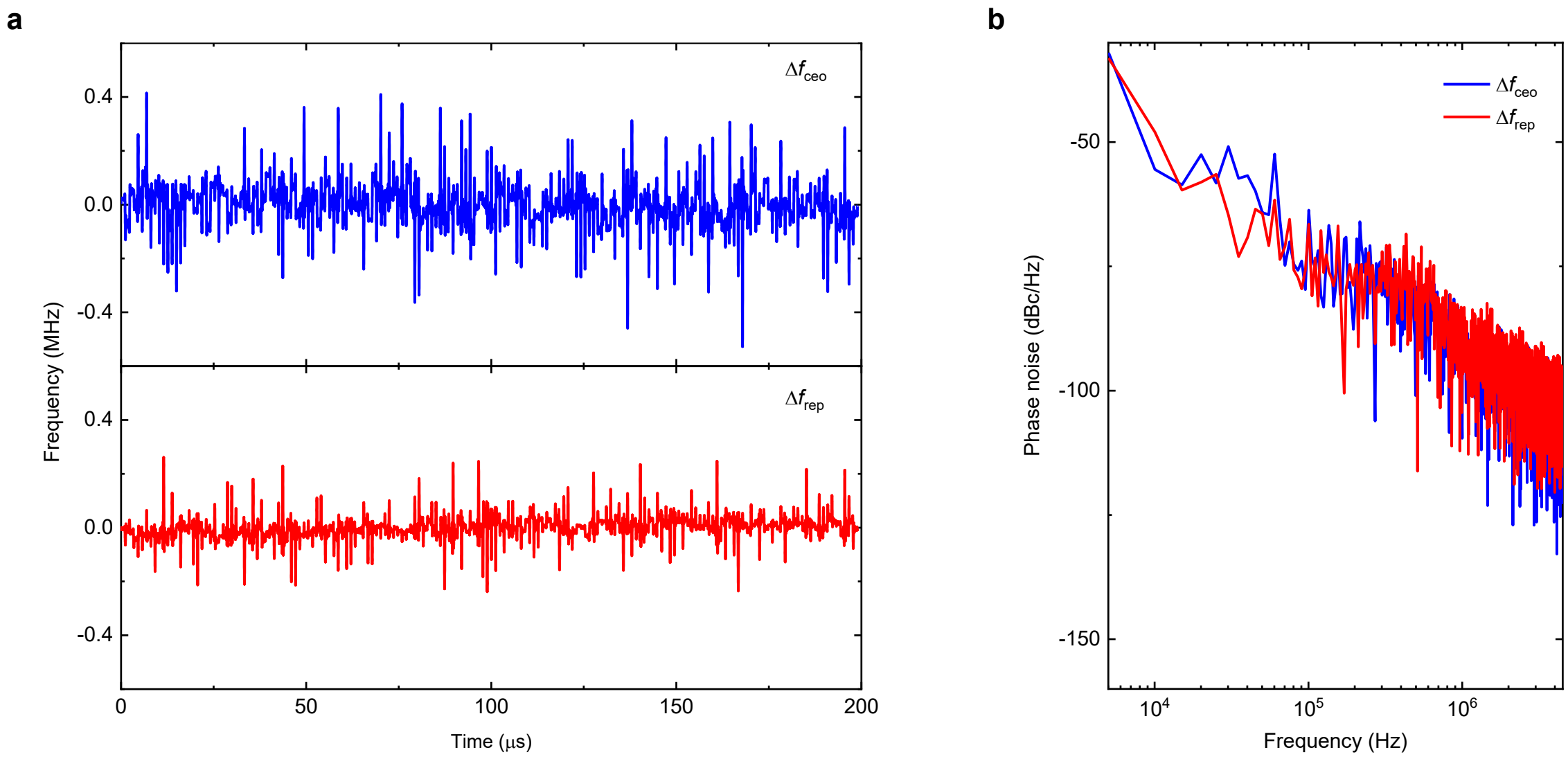

Figure S5: Results obtained from a single-shot acquisition window of 200 μs for the fundamental–second-harmonic dual-comb state. **a** Instantaneous frequency-fluctuation traces extracted from the raw dual-comb time-domain interferogram, where the upper trace corresponds to the carrier-envelope offset frequency difference Δf_{ceo} and the lower trace corresponds to the repetition frequency difference Δf_{rep}. **b** Corresponding single-sideband phase noise spectra calculated from the extracted frequency fluctuations.

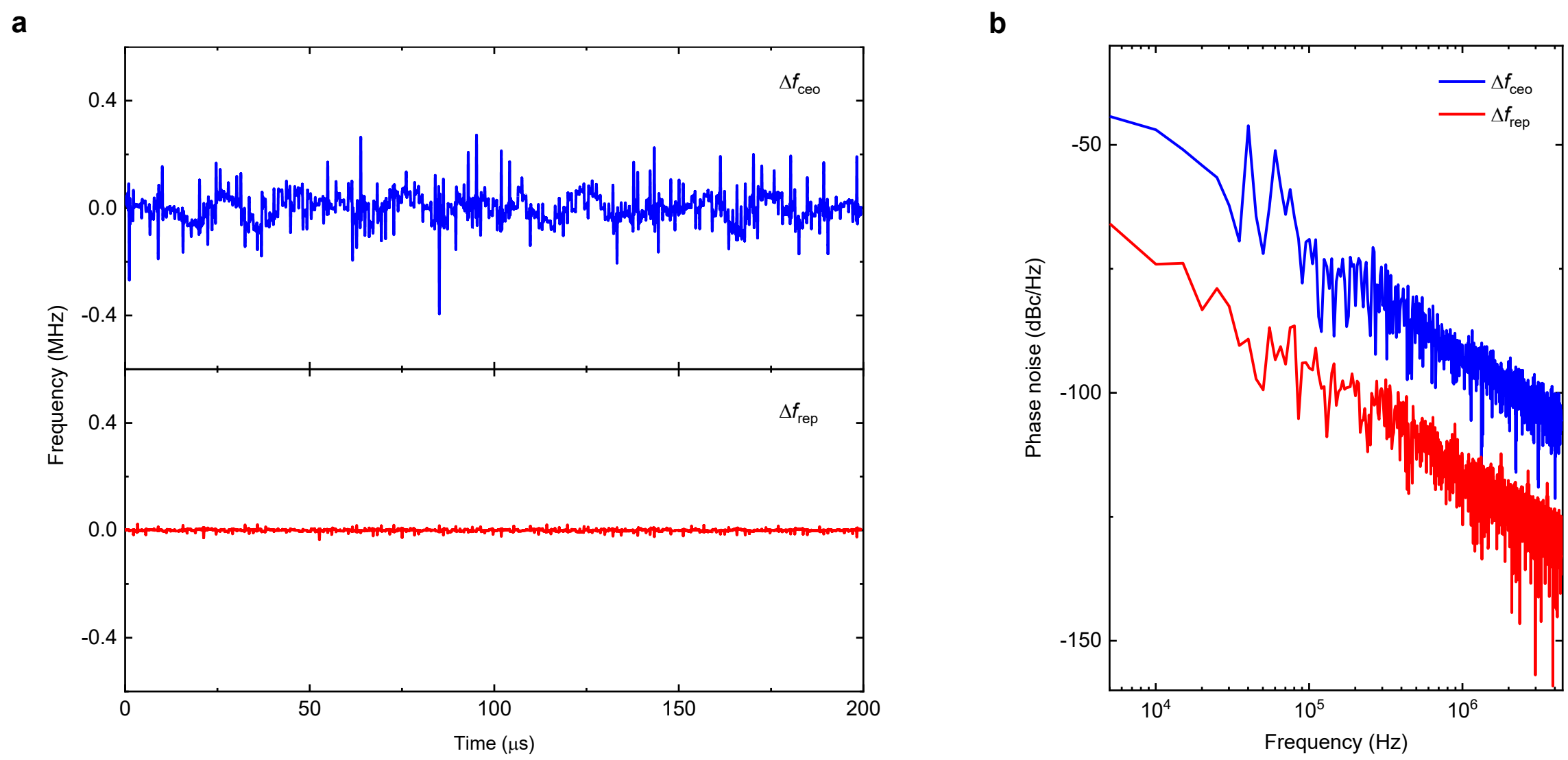

Figure S6: Results obtained from a single-shot acquisition window of 200 μs for the second-harmonic – second-harmonic dual-comb state. **a** Instantaneous frequency-fluctuation traces extracted from the raw dual-comb time-domain interferogram, where the upper trace corresponds to the carrier-envelope offset frequency difference Δf_{ceo} and the lower trace corresponds to the repetition frequency difference Δf_{rep}. **b** Corresponding single-sideband phase noise spectra calculated from the extracted frequency fluctuations.

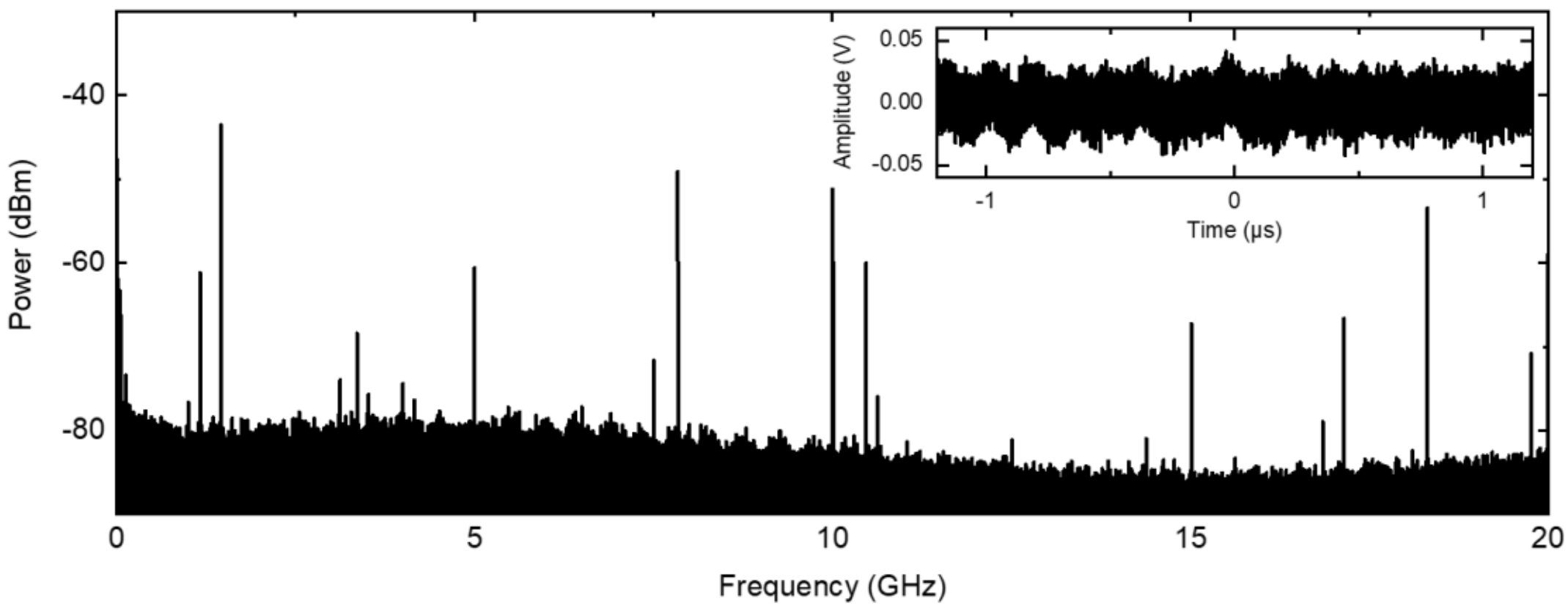

Figure S7: Radio-frequency dual-comb spectrum of the second-harmonic–third-harmonic dual-comb state, obtained by applying a fast Fourier transform (FFT) to the time-domain dual-comb signal. The oscilloscope sampling rate is 40 GSa/s. The main panel shows the spectrum retrieved from a single-shot, unaveraged time trace recorded over an acquisition window of 200 μs. The inset shows a magnified view of the time-domain trace over a 2.5 μs interval. The positions of the FFT-retrieved dual-comb modes are in good agreement with those measured by the spectrum analyzer in Figure 6 of the main text, confirming the consistency between the two measurement approaches. The additional spurious features mainly originate from the internal clock of the oscilloscope, electronic background, and environmental noise, rather than from the intrinsic multi-heterodyne dual-comb signal.
